# Development and application of a rapid all-in-one plasmid CRISPR-Cas9 system for iterative genome editing in *Bacillus subtilis*

**DOI:** 10.1186/s12934-022-01896-0

**Published:** 2022-08-23

**Authors:** Yu Zou, Lu Qiu, Aowen Xie, Wenyuan Han, Shangbo Zhang, Jinshan Li, Shumiao Zhao, Yingjun Li, Yunxiang Liang, Yongmei Hu

**Affiliations:** 1 State Key Laboratory of Agricultural Microbiology, College of Life Science and Technology, Huazhong Agricultural University, Wuhan, 430070 People’s Republic of China; 2 Bioengineering Center, College of Life Science and Technology, Huazhong Agricultural University, Wuhan, 430070 China

**Keywords:** *Bacillus subtilis*, PAMPL, All-in-one system, Iterative genome editing, *Douchi* fibrinolytic enzyme

## Abstract

**Background:**

*Bacillus subtilis*, an important industrial microorganism, is commonly used in the production of industrial enzymes. Genome modification is often necessary to improve the production performance of cell. The dual-plasmid CRISPR-Cas9 system suitable for iterative genome editing has been applied in *Bacillus subtilis*. However, it is limited by the selection of knockout genes, long editing cycle and instability.

**Results:**

To address these problems, we constructed an all-in-one plasmid CRISPR-Cas9 system, which was suitable for iterative genome editing of *B. subtilis*. The PEG4000-assisted monomer plasmid ligation (PAMPL) method greatly improved the transformation efficiency of *B. subtilis* SCK6. Self-targeting sgRNA_rep_ transcription was tightly controlled by rigorous promoter P_acoR_, which could induce the elimination of plasmids after genome editing and prepare for next round of genome editing. Our system achieved 100% efficiency for single gene deletions and point mutations, 96% efficiency for gene insertions, and at least 90% efficiency for plasmid curing. As a proof of concept, two extracellular protease genes *epr* and *bpr* were continuously knocked out using this system, and it only took 2.5 days to complete one round of genome editing. The engineering strain was used to express *Douchi* fibrinolytic enzyme DFE27, and its extracellular enzyme activity reached 159.5 FU/mL.

**Conclusions:**

We developed and applied a rapid all-in-one plasmid CRISPR-Cas9 system for iterative genome editing in *B. subtilis,* which required only one plasmid transformation and curing, and accelerated the cycle of genome editing. To the best of our knowledge, this is the rapidest iterative genome editing system for *B. subtilis*. We hope that the system can be used to reconstruct the *B. subtilis* cell factory for the production of various biological molecules.

**Supplementary Information:**

The online version contains supplementary material available at 10.1186/s12934-022-01896-0.

## Background

Clustered regularly interspaced short palindromic repeat (CRISPR) and CRISPR-associated genes (Cas) function as adaptive immune systems in 90% of archaea and 40% of bacteria [1, 2]. The systems employ RNA-guided nucleases to specifically recognize and cleave viral DNA to perform the immunity. The feature endows them the ability to cleave any desired sites in genome and thus the potential to be repurposed as genome editing tools [3, 4]. The most widely applied system is CRISPR-Cas9. Cas9 endonuclease recognizes the target site in the genome through the 20 bp nucleotide at the 5' end of single-guide (sg) RNA and generates DNA double-strand break (DSB) [5]. DSB can be repaired by random insertion or deletion caused by non-homologous end joining (NHEJ), or by homologous recombination (HR) to achieve precise repair [6]. Since most bacteria lack the NHEJ pathway, DSB caused by Cas9 nuclease is lethal to bacteria [7]. Therefore, CRISPR-Cas9 can be used as a reverse selection marker to effectively kill cells that have not been edited, and it can also improve the efficiency of genome editing [8].

*Bacillus subtilis* has been not only widely used for the production of medicines and cosmetics, but also used as an ideal expression host for various industrial enzymes, such as amylase, cellulose and protease [9–13]. In recent years, the CRISPR-Cas9 system has been used in *B. subtilis* genome editing by plasmids carrying CRISPR elements. However, the plasmid must be removed before the next round of genome editing. Plasmid curing and transformation are time-consuming, especially in iterative genome editing. Designing an inducible promoter to control the transcription module of sgRNA with self-targeting function can be used to cure plasmids and accelerate the genome editing cycle. Currently, the existing CRISPR-Cas9 system suitable for iterative genome editing of *B. subtilis* is dual-plasmid system. Using the spore-related promoter P_spo0A_ to control the transcription of sgRNA with self-targeting function can achieve the purpose of spontaneously curing the plasmid after genome editing. However, the dual-plasmid system still has some limitations, such as the inability to edit genes related to σ^E^, which limits its application in metabolic engineering and synthetic biology [14]. Compared with dual-plasmid system, all-in-one plasmid system has the advantages of higher stability and lower metabolic burden on the host [15]. Moreover, all-in-one plasmid CRISPR-Cas9 system only requires one plasmid transformation and curing, so it has more advantages in iterative genome editing.

In this study, we constructed an all-in-one CRISPR-Cas9 system for iterative genome editing in *B. subtilis*. In addition, we developed a new method for the formation of multimeric plasmids. This method was called PEG4000-assisted monomeric plasmid ligation (PAMPL), which significantly improved the transformation efficiency of *B. subtilis* SCK6. Furthermore, the CRISPR-Cas9 system could efficiently and rapidly introduce various types of genomic modifications, including point mutation, insertion and deletion. It only takes 2.5 days to complete one round of genome editing cycle. At last, as a proof of concept, this all-in-one system was used to construct *B. subtilis* cell factory suitable for protein production.

## Results

### Design and construction of an all-in-one plasmid CRISPR-Cas9 system with self-curing module

**Fig. 1:**
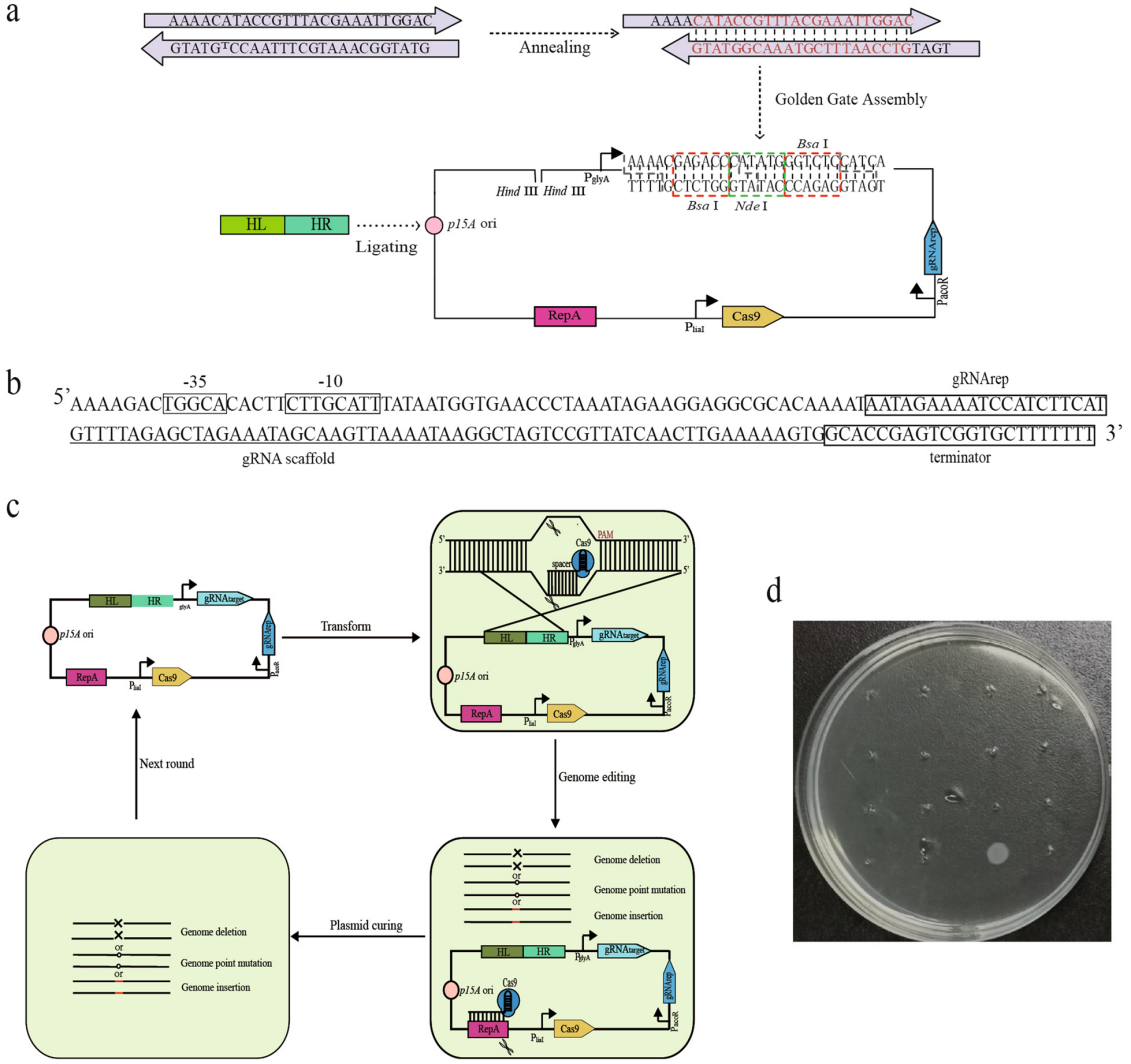
Plasmid design and construction of the all-in-one plasmid CRISPR-Cas9 system. **a** Assembly of spacer and donar DNA. The gRNA_target_ under the constitutive promoters for genome editing. The another gRNA_rep_ under the inducible promoter for plasmid curing. **b** Squence of gRNA_rep_ under the control of P_acoR_ promoter. **c** Strategy for iterative genome editing in *B. subtilis*. In the genome editing phase, sgRNA_target_/Cas9 complex cuts the genome for homologous recombination. In the plasmid curing phase, sgRNA_rep_/Cas9 complex tragets the *repA* gene to eliminate the editing plasmid. **d** Tetracycline sensitivity test for the plasmid self-curing system, 16 clones were picked for tetracycline sensitivity test, and only one single clone showed tetracycline resistance

Iterative genome editing (IGE) requires to efficiently cure the genome editing plasmid, and targeting replicons or resistance genes of the plasmid using a gRNA has been employed for this purpose [14, 16–19]. In this study, we constructed an all-in-one plasmid that utilizes CRISPR-Cas9 system for IGE in *B. subtilis*. This plasmid contains a *Cas9* gene expression cassette and expresses gRNA_rep_ that targets the replicon of the plasmid (Fig. 1a). The spacer that will guide the cleavage at the targeted gene (gRNA_target_) and donor DNA can be inserted into the plasmids at the designed restriction sites (Fig. 1a). Expression of gRNA_rep_ is controlled by a rigorous acetoin-inducible promoter which has no basal expression level in the presence of phosphotransferase system (PTS) sugar (Fig. 1b). The constitutive promoter P_glyA_ was used to express gRNA_target_ [20]. In order to ensure the orthogonality of the promoters, we chose the non-carbohydrate bacitracin inducible promoter P_liaI_ to express the Cas9 nuclease [21]. To reduce the possibility of internal recombination of the two gRNA expression cassettes, we reversed the transcription direction of the expression cassette gRNA_rep_ and gRNA_target_ [22]. Together, the all-in-one plasmid allows sequential genome editing and plasmid curing. After the plasmid is transferred into cells, the expression of Cas9 will be induced and it will cleave genomic DNA at the designed sites as guided by the constitutively expressed gRNA_target_. The cleaved DNA will be repaired by homologous recombination repair using the donor DNA to obtain desired mutations. At last, gRNA_rep_ will be induced to cure the plasmid and the resulting plasmid-free cells will be suitable for next round genome editing (Fig. 1c). We picked 16 clones for tetracycline sensitivity test, and the results showed that only one single clone showed tetracycline resistance, indicating that the plasmid self-curing system could work efficiently (Fig. 1d).

### Effective transformation method for high molecular weight plasmids

To analyze the genome editing efficiency of the strategy, we constructed two knockout plasmids, pGE-KOerm and pGE-KOamyE, which are supposed to target *erm* and *amyE* in the *B. subtilis* SCK6 genome respectively. However, we found that no clone was obtained when pGE-KOerm was introduced to *B. subtilis* SCK6 at first. This was probably due to the large size (11.0 kb) of plasmid. *B. subtilis* prefer to uptake multimeric plasmids rather than monomeric plasmids during competent phase. This might be because the monomeric plasmid would be degraded after entering the cell, and the intramolecular recombination occurred after the multimeric plasmid enter the cells would cause the plasmids to circularize [23]. However, the multimeric plasmid formed by "simple clone" method is limited by its large size of plasmid which inevitably introduces unnecessary mutations in the PCR, and it is difficult to anneal and complement the overlapping sequences at both ends of PCR products effectively [24].

**Fig. 2:**
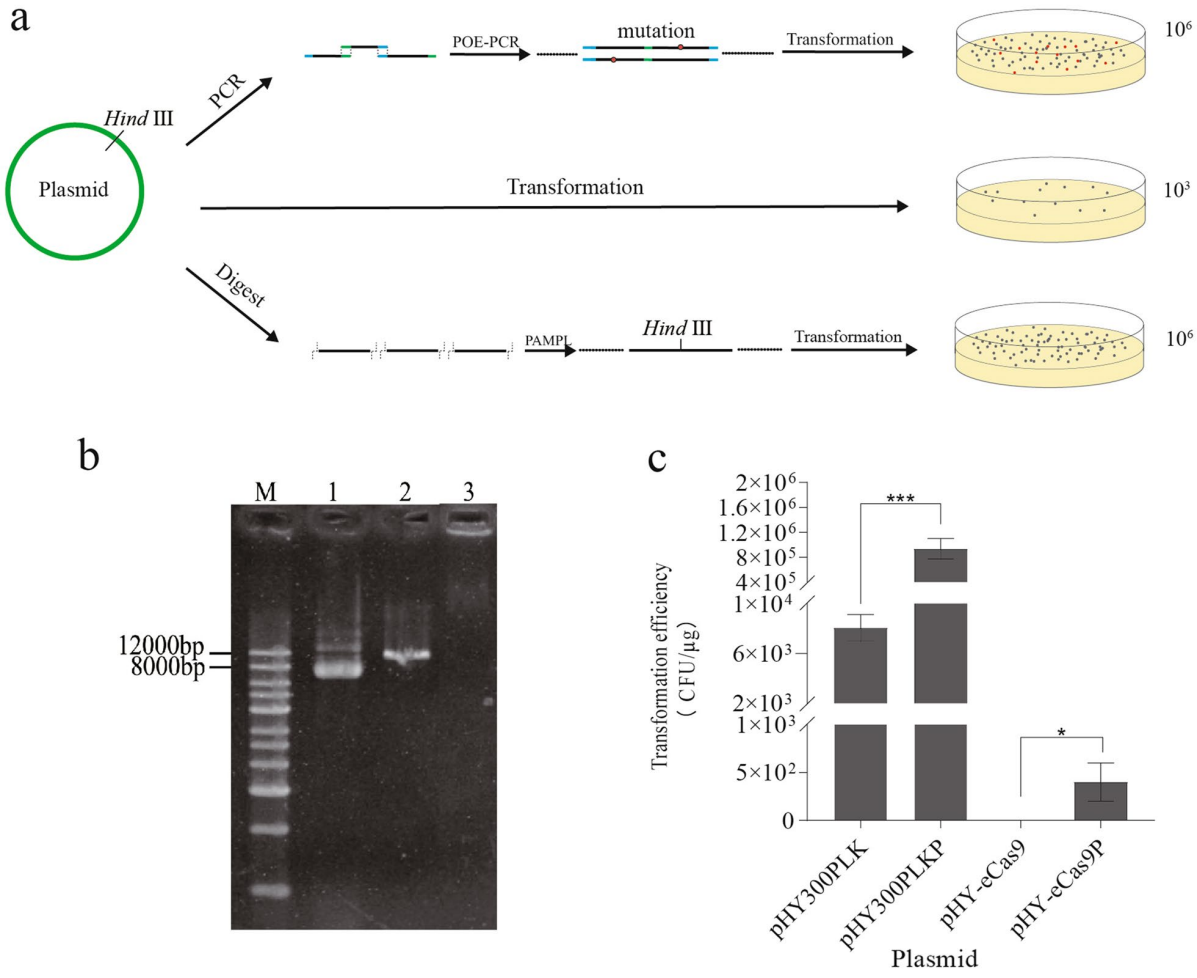
Enhancing transformation efficiency through PAMPL method. **a** Different transformation methods for *B. subtilis* SCK6. PCR inevitably introduce unnecessary mutations and it is not suitable for large-sized plasmid. POE method requires a high concentration of monomeric plasmid to ensure the formation of multimeric plasmid. Direct transformation of monomeric plasmids into *B. subtilis* SCK6 is inefficient. The PAMPL method we exploited could improve the transformation efficiency of *B. subtilis* SCK6 by using PEG4000. **b** Gel electrophoresis of different types of CRISPR-Cas9 plasmid pGE-KOerm. M: 1 kb Marker; 1. monomeric plasmid; 2. linearized plasmid; 3. multimeric plasmid. **c** Transformation efficiency of different types of plasmids in *B. subtilis* SCK6. *Represent *p* < 0.05, ***represent *p* < 0.001

Therefore, we used the PEG4000-assisted monomeric plasmid ligation (PAMPL) method to make multimeric plasmids from monomeric plasmids. PEG4000 is a molecular coagulant, which can greatly promote intermolecular connection and inhibit intramolecular connection. In the presence of PEG4000, linear plasmids could be ligated into multimeric plasmids without high concentration (Fig. 2a). The multimeric plasmids with branched structure were high molecular weight products so that they cannot migrate into the gel (Fig. 2b).

The monomeric plasmids pHY300PLK, pGE-KOerm and correspond multimeric plasmids pHY300PLKP, pGE-KOermP were transformed into *B. subtilis* SCK6. We found that the transformation efficiency of the plasmid pHY300PLK increased from 8 × 10^3^ to 1 × 10^6^ CFU/μg, and the transformation efficiency of the knockout plasmid pGE-KOermP increased from 0 to 3 × 10^2^ CFU/μg (Fig. 2c).Thus, the results indicated that PAMPL method could improve the transformation efficiency, which provided a convenient strategy for transformation of large size plasmids *into B. subtilis* SCK6.

### The genome editing efficiency of the all-in-one plasmid

**Fig. 3:**
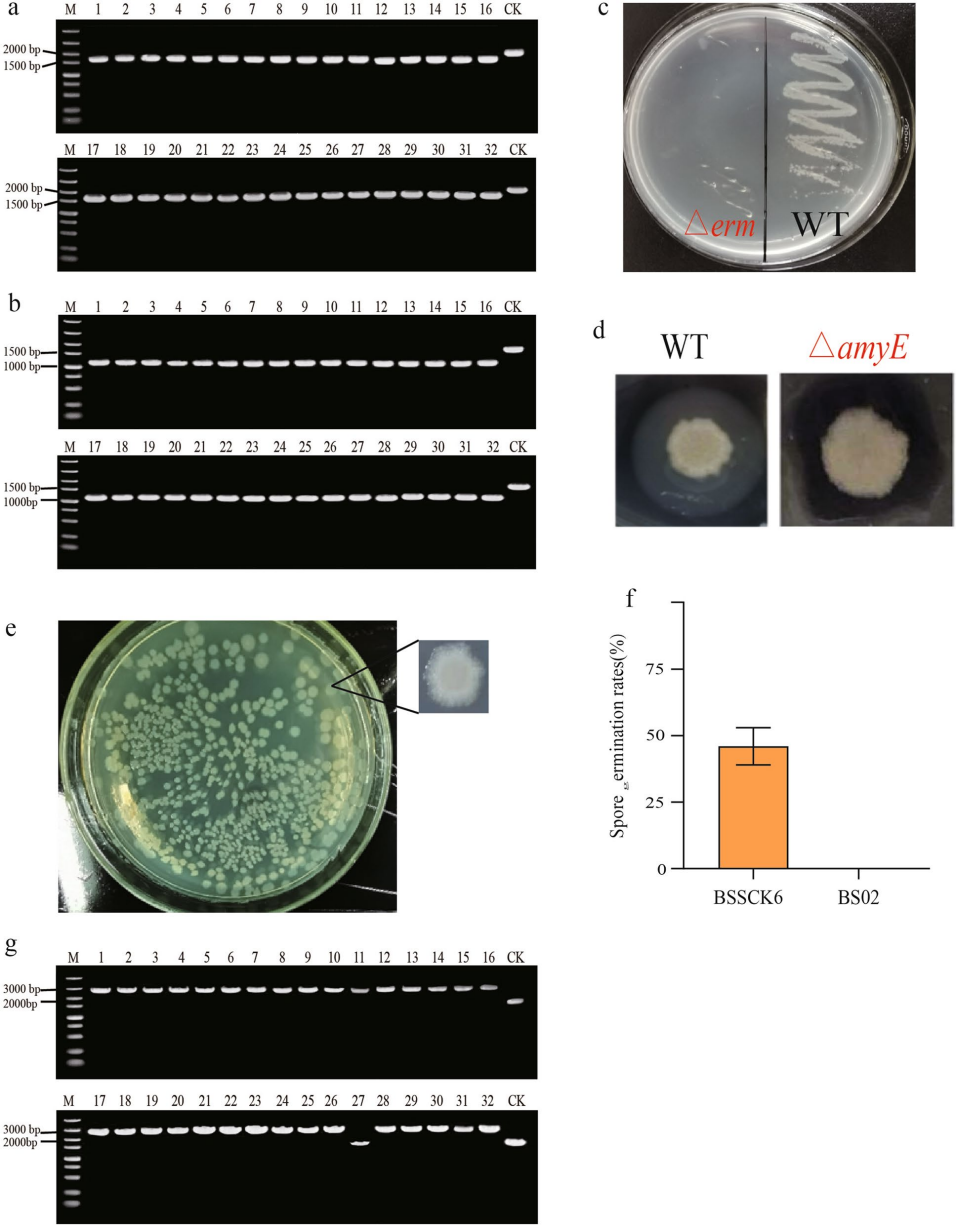
The genome editing efficiency of the all-in-one plasmid. **a** PCR verification of *erm* gene knockout. Lanes 1–32 represent the PCR product of mutant (1583 bp), respectively. All 32 clones were shown to harbor the desired deletion by colony PCR; CK represents the PCR product of wild-type strain (1949 bp). **b** PCR verification of *amyE* gene knockout. Lanes 1–32 represent the PCR product of mutant (1114 bp), respectively. All 32 clones were shown to harbor the desired deletion by colony PCR; CK represents the PCR product of wild-type strain (1614 bp). **c** Phenotypic verification of *erm* gene knockout. The wild-type strain grew well on the erythromycin plate, while △*erm* mutant strain was sensitive to erythromycin due to the knockout of the erythromycin gene, resulting in inhibited growth on the plate. **d** Phenotypic verification of *amyE* gene knockout. The wild-type strain has obvious hydrolysis ring, while the △*amyE* mutant strain displayed no transparent ring in the starch-plate. **e** Phenotypic verification of the point mutation of *spo0A* gene. The colony morphology of *spo0A*^A714T^ mutant strain was more transparent. **f** Sporulation germination rates of the wild-type strain BSSCK6 and the *Spo0A* point mutation strain BS02. **g** PCR verification of the *egfp* insertion efficiency. Lanes 1–32 represent the PCR product of mutant (2711 bp), respectively. CK represents the PCR product of wild-type strain (1614 bp)

With PAMPL, we obtained the transformants of pGE-KOerm and pGE-KOamyE. And then evaluated the single gene knock out efficiency of all-in-one plasmid using *erm* and *amyE* gene as examples, respectively (Additional file 1: Figs. S1, S2). We picked 32 clones and all clones were shown to harbor the desired deletion by colony PCR (Fig. 3a, b). In addition, The △*erm* mutant strain was sensitive to erythromycin and the another △*amyE* mutant strain displayed no transparent ring in starch-plate (Fig. 3c, d). The results showed that the single gene knockout efficiency of the all-in-one plasmid reached 100%, demonstrating high efficiency of the all-in-one plasmid for single gene deletions (Table 1).

**Table 1:** Summary of genome editing efficiency and plasmid curing efficiency of CRISPR-Cas9 system

Plasmid	CFU(counts/mL)	Editing efficiency	Curing efficiency
pGE-KOerm	87 ± 7	32/32 (100%)	46/48 (95.8%)
pGE-KOamyE	42 ± 2	32/32 (100%)	44/48 (91.6%)
pGE-PMspo0A	660 ± 42	660/660 (100%)	47/48 (97.9%)
pGE-Iegfp	94 ± 4	30 ± 1/32 (93.8% ± 3.1%)	46/48 (95.8%)

The CFU (counts/mL) and editing efficiency (in the parentheses) are given as mean ± SD of three independent studies

Next, we examined the applicability of the all-in-one plasmid to introduce point mutation into *spo0A* gene. The *spo0A* is principal transcription factor that controls the first stage of spore formation. We converted adenine at the 714-position of *spo0A* into thymine, which would result in a stop codon to terminate translation in advance (Additional file 1: Fig. S3a). It was found that the colony morphology of *spo0A*^A714T^ mutant strain was more transparent (Fig. 3e). Furthermore, the *spo0A*^A714T^ mutant strain was cultured in DSM medium (sporulation medium) but failed to form spores (Fig. 3f). The sequencing results confirmed that we obtained the desired mutation and that the mutation efficiency was 100% (Additional file 1: Fig. S3b and Table 1). Subsequently, the insertion was also tested by integration an EGFP cassette into genome (Additional file 1: Fig. S4). The results show that CRISPR-Cas9 system produce 94% insertion efficiency (Fig. 3g and Table 1). Finally, we used the curing module to eliminate the parent plasmid. The curing efficiency of all plasmids reached at least 90% (Table 1). The results indicate that the all-in-one plasmid could be used for versatile genome modification with high efficiency rapidly.

### IGE using the all-in-one plasmid strategy

**Fig. 4:**
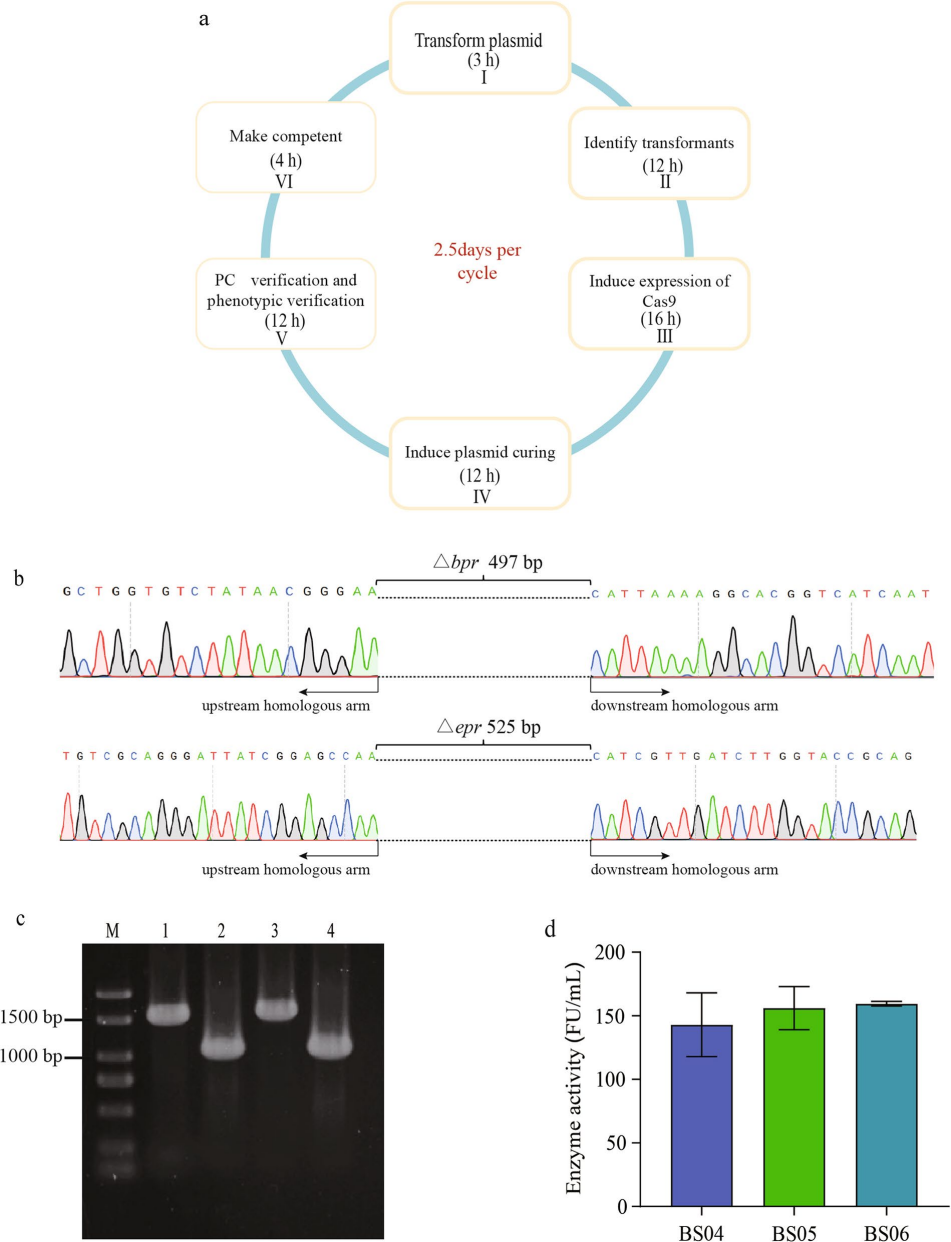
The application of CRISPR-Cas9 system for genome engineering in *B. subtilis.* **a** Diagram of the iterative genome editing procedure. **b** Sanger sequencing analysis of integrated site. **c** Colony PCR was performed to verify the knockout of *epr* gene and *bpr* gene. Lane 1: The PCR product of wild type (1551 bp); Lane 2: The PCR product of mutant type (1040 bp); Lane 3: The PCR product of wild type (1517 bp); Lane 4: The PCR product of mutant type (1008 bp). **d** Effect of diverse hosts on *Douchi* fibrinolytic enzyme production

Since the all-in-one plasmid CRISPR-Cas9 system had high genome editing efficiency (Table 2), nearly 100% cells in the culture after Cas9 induction should carry the desired mutations. Therefore, we did not verify the culture but directly subjected it to plasmid curing to further save time during IGE. Theoretically, it only takes 2.5 days for the all-in-one plasmid strategy to complete a genome editing cycle during IGE (Fig. 4a).

**Table 2:** Strains and plasmids used in this study

Strains or plasmids	Relevant properties	Source of references
Strains		
* E. coli* DH5α	F-φ80*lac*ZΔM15Δ(*lac*ZYA-*ar*gF)Μ169*en*A1*rec*A1*hsd*R17(*rk*-,*mk*-)*sμp*E44λ-*thi*-1gyrA96relA1*pho*A	WeiDi (Shang hai)
* B. subtilis* SCK6	Δ*his*, Δ*npr*R*n*, Δ*npr*E18, *lac*A::P_*xylA*_-*com*K, *apr*A3 *egl*S102, *bgl*T, *bgl*SRV, E*rm*^R^	Laboratory preservation
BS01	*B. sublitis* SCK6 derivative with its *amyE* deleted	This study
BS02	*B. sublitis* SCK6 derivative with its *spo0A* deleted	This study
BS03	BS01 derivative with *epr* and *bpr* deleted	This study
BS04	*B. subtilis* SCK6 + pHY-SDFE27	This study
BS05	BS02 + pHY-SDFE27	This study
BS06	BS03 + pHY-SDFE27	This study
Plasmids		
pHY300PLK	*E. coli-B. subtilis* shutter plasmid, Tet^R^, Amp^R^	Laboratory preservation
pCRISPomyces-2	Streptomyces expression of codon-optimized Cas9 and custom gRNA	addgene#61737
pHY-ngCas9	pHY300PLK derivative, P_glyA_-*Bsa*I-spacer-*Bsa*I-gRNAscffold-T1 terminator, P_acoR_-gRNA_rep_, P_liaI_-Cas9	This study
pGE-KOerm	pHY-ngCas9 derivative, 20 bp gRNA targeting *erm*, 1000 bp donor DNA(deletion *erm*)	This study
pGE-KOamyE	pHY-ngCas9 derivative, 20 bp gRNA targeting *amyE*, 1000 bp donor DNA(deletion *amyE*)	This study
pGE-PM*spo0A*	pHY-ngCas9 derivative, 20 bp gRNA targeting *spo0A*, 1000 bp donor DNA(point mutantion *spo0A*)	This study
pGE-I*egfp*	pHY-ngCas9 derivative, 20 bp gRNA targeting *amyE*, 1500 bp donor DNA(insetion P_*43*_*-egfp-*T1 terminator)	This study
pGE-KObpr	pHY-ngCas9 derivative, 20 bp gRNA targeting *bpr*, 1000 bp donor DNA(deletion *bpr*)	This study
pGE-KOepr	pHY-ngCas9 derivative, 20 bp gRNA targeting *epr*, 1000 bp donor DNA(deletion *epr*)	This study
pHY-SDFE27	pHY300PLK derivative, P_spovg_-DFE27-T1 terminator	This study

As a proof of concept, we used the strategy to continuously knock out two extracellular protease genes *epr* and *bpr* of the strain BS01, generating the strains BS02 and BS03 respectively (Fig. 4b, c). The strains could be suitable to express extracellular proteins. We chose to express the *Douchi* fibrinolytic enzyme. The enzyme exhibits high thrombolytic activity and it has great application prospects in the prevention and treatment of cardiovascular and cerebrovascular diseases [25].

The *Douchi* fibrinolytic enzyme expression vector pHY-SDFE27 was transformed into strains *B. subtilis* SCK6, BS02 and BS03 to obtain the strains BS04, BS05, and BS06 respectively. The strains BS04, BS05 and BS06 were fermented in a shake flask with TB medium for 30 h, and the extracellular enzyme activities in the supernatant of the culture were measured to be 143.0 FU/mL, 156.0 FU/mL and 159.5 FU/mL, respectively. The enzyme activity in the culture supernatant of the strains BS02 and BS03 increased by 9% and 11.5% respectively, compared with the wild-type strain *B. subtilis* SCK6 (Fig. 4d).

## Conclusions

Here, we developed and applied a rapid all-in-one plasmid CRISPR-Cas9 system for iterative genome editing in *B. subtilis* by constructing P_acoR_-sgRNA_rep_ self-curing module. This system can achieve 100% single genome knockout efficiency, 100% point mutation efficiency and 96% gene insertion efficiency, and it only takes 2.5 days to complete one round of genome editing. It shows that it has the advantages of high efficiency and rapidity in genome editing. Then, two extracellular proteases *bpr* and *epr* on BS01 were knocked out and the engineered strain produced 159.5 FU/mL of *Douchi* fibrinolytic enzyme activity. The methods will boost the improvement of *B. subtilis* chassis cells for producing bio-enzymes.

## Methods

### Strains and culture conditions

All strains and plasmids used in this study were listed in Table 2. *Escherichia coli* DH5α was used as the host for plasmid construction. *E. coli* and *B. subtilis* were cultured at 37 °C, in Luria–Bertani (LB) medium (10 g/L peptone, 5 g/L yeast extract and 10 g/L NaCl), with shaking at 220 r/min. Plasmid curing was performed in arabinose limitation medium (ALM), containing 50 mM Tris, 15 mM (NH_4_)_2_SO_4_, 8 mM MgSO_4_, 27 mM NaCl, 7 mM sodium citrate, 0.4 mM KH_2_PO_4_, 2 mM CaCl_2_, 1 µM FeSO_4_, 10 µM MnSO_4_, 4.5 mM sodium glutamate, 0.1% arabinose, 160 μg/mL histidine, pH 7.5. Fermentation of *Douchi* fibrinolytic enzyme was performed in terrific broth (TB) medium, containing 24 g/L yeast extract, 12 g/L peptone, 5 g/L glycerol, 1.8 mM CaCl_2_, 17 mM K_2_HPO_4_ and 72 mM KH_2_PO_4_. Spore formation in Difco nutrient broth sporulation medium (DSM), containing 3 g/L beef extract, 5 g/L peptone, 13 mM KCl, 0.002 mM MnCl_2_, 0.5 mM CaCl_2_, 0.001 mM FeSO_4_·7H_2_O, pH 7.0.

When required, 100 μg/mL ampicillin, 20 μg/mL tetracycline, and 1 μg/mL erythromycin were supplied into the medium. Zinc bacitracin (50 μg/mL) was supplemented to induce the expression of Cas9, while 1% glucose was used to inhibit the transcription of gRNA_rep_. For plasmid curing, acetoin (0.1%) was added to induce gRNA_rep_. Agar was added at 15 g/L to prepare solid medium.

### Plasmid construction

The primers used in this study were shown in Additional file 1: Table S1. Point mutation method was used to remove the two *Bsa* I restriction sites from the plasmid pHY300PLK. Using *B. subtilis* SCK6 genome and pCRISPomyces-2 as templates, P_liaI_ promoter and *cas9* gene were amplified respectively. P_glyA_-*Bsa* I-*Nde* I- *Bsa* I-gRNA scaffold-T1 terminator and P_acoR_-gRNA_rep_-gRNA scaffold fragments were synthesized by GenScript (Piscataway, NJ). All the above fragments were linked to the vector pHY300PLK by T4 enzyme ligation and seamless cloning to form pHY-ngCas9. Bacitracin-inducible promoter P_liaI_, constitutive promoter P_glyA_ and acetoin-inducible promoter P_acoR_ were used for the expression of *cas9* gene, gRNA_target_ and gRNA_rep_, respectively. The upstream and downstream homology arms used for genome editing were amplified using *B. subtilis* SCK6 genome as a template. The 20 bp spacer sequence targeting the genome was synthesized by annealing oligonucleotides. To obtain the plasmid pGE-KOerm, pGE-KOamyE, pGE-PMspo0A, pGE-Iegfp, pHY-KObpr and pHY-KOepr, the homologous recombination template and 20 bp spacer sequence were respectively linked to pHY-ngCas9 by restriction enzyme digestion with *Hind* III, ligation and Golden Gate Assembly (Fig. 1a).

### Transformation of *B. subtilis* SCK6

Plasmids were introduced into *B. subtilis* SCK6 by transformation as described by Zhang with some modifications [16]. The plasmid extracted from *E. coli* DH5α was linearized by the restriction enzyme *Xba* I, and heated at 65 °C for 20 min to inactivate the restriction enzyme *Xba* I. To convert 200 ng of linearized plasmid into multimeric plasmid, 15% PEG4000 was added to the enzyme-linked system, and then 100 ng of the multimeric plasmid was transformed into 500 μL competent cells.

### Genome editing

The knockout plasmid was transformed into *B. subtilis* SCK6 and the transformants were picked and inoculated into LB liquid medium supplemented with 20 µg/mL tetracycline. When the culture OD_600_ reached 0.05, the culture solution was supplemented with 50 µg/mL zinc bacitracin and 1% glucose, and the bacterial strains were induced for 16 h. The culture was spread onto LB plates for 12 h. The mutations were confirmed by phenotypic validation, PCR and DNA sequencing. To cure plasmid, the positive clones were picked and cultured for 16 h with ALM liquid medium containing 0.1% acetoin and 50 µg/mL zinc bacitracin. And then the culture was spread onto LB plates. The plasmid-cured mutants were obtained by tetracycline selection and further verified through PCR. The editing efficiency and curing efficiency was determined by calculating the PCR analytical number of positive colonies.

To further accelerate the genome editing cycle, the induced bacterial solution was transferred at a rate of 1‰ to ALM medium containing 0.1% acetoin and 50 µg/mL zinc bacitracin, and then induced for 16 h. The tetracycline sensitive positive clones were obtained by spreading the bacterial solution on the LB plate after a series of dilutions. Subsequently, the positive clones were further verified by PCR to confirm whether the gene was iteratively edited.

### Sporulation assays

The overnight seed cultures were inoculated into DSM liquid culture and cultivated at 37 °C with vigorous aeration for approximately 24 h, and then heated to 80 °C for 20 min. Serial dilutions were prepared with each culture using sterile water, and 100 μL of the culture was spread onto LB plates. Viable colony-forming units were evaluated after overnight incubation at 37 °C. The activated culture of the strain was inoculated into DSM spore-forming medium for 24 h.

### Determination of fibrinolytic activity

The enzyme activity of *Douchi* fibrinolytic enzyme was determined according to the Japan Natto Kinase Association (http://j-nattokinase.org/jnka_nk_english.htmL). In brief, 0.2 mL of fibrinogen (bovine, Yuanye, Shanghai, China) solution (0.72%, w/v) was first mixed with 0.7 mL of Tris–HCl buffer (50 mM, pH 8.0). After incubation at 37 °C for 10 min, 50 μL of thrombin (20 U/mL) was added to the reaction solution. After thorough blending, the mixture was incubated at 37 °C for 10 min. Subsequently, 100 μL of fermentation supernatant with appropriate dilution was supplemented into the catalytic solution. After 60 min, 1 mL of trichloroacetic acid (TCA, 200 mM) was added the catalytic solution to terminate the reaction followed by water bath at 37 °C for 20 min. The supernatant containing *Douchi* fibrinolytic enzyme was obtained by centrifuging the fermentation broth at 12,000 r/min for 15 min, and then the absorbance at 275 nm was measured with an ultraviolet–visible-near infrared (UV–vis-NIR) spectrophotometer (UV-3600, SHIMADZU). All data are the average of three independent samples. One unit of *Douchi* fibrinolytic enzyme activity was defined as the amount of enzyme required to produce a change of 0.01 per minute at 37 °C, pH 8.0.

### Statistical analysis

All data were presented as the mean ± standard deviation (SD) from at least three independent experiments. The t-test was used to detect differences and significance levels were: *P < 0.05, **P < 0.01, ***P < 0.001.

## Discussion

Recently, several CRISPR-Cas9-based genome editing systems in *B. subtilis* have been reported, including integrated systems, all-in-one systems, and dual-plasmid systems. The chromosome-integrated system has a knockout efficiency of 100% for single genes, 85% for double genes, and a insertion efficiency of 69% for a 2.9 kb hyaluronic acid synthesis gene. However, the problem with the integrated system was that since the Cas9 gene was integrated into the genome of the host bacteria, it could not be completely knocked out without leaving any trace of foreign DNA on the genome [26]. Although Zhang et al. constructed an all-in-one plasmid CRISPR-Cas9 system and knocked out 5 genes several times, the knockout efficiency of the system was only between 35 and 55% [27]. Compared with all-in-one plasmid CRISPR-Cas9 system, dual-plasmid CRISPR-Cas9 system has higher assembly and editing efficiency. So et al. constructed a dual-plasmid CRISPR-Cas9 system, which achieved a single gene knockout efficiency of 100%, a site-directed mutation efficiency of 68%, and a large genome fragment knockout efficiency of 80% [28]. However, when performing iterative genome editing, the common removing method of knockout plasmids is still time-consuming. In this study, we constructed an all-in-one CRISPR-Cas9 system for iterative genome editing in *B. subtilis*. Due to the rational element design, our system achieved 100% efficiency for single gene deletions and point mutations, 96% efficiency for gene insertions, and above 90% efficiency for plasmid curing. Furthermore, compared with the dual-plasmid CRISPR-Cas9 system with self-curing modules, our all-in-one system has fewer transformation steps and could complete one round of genome editing in just 2.5 days.

In previous studies, *B. subtilis* could not carry monomeric plasmids because single-stranded DNA would be degraded in the absence of other plasmids to pair with [29], resulting in the low efficiency during transformation and subsequent genome editing, which limited the CRISPR-Cas9 system [30]. However, if the plasmid enters the competent cell in the form of a multimer, the transformation efficiency can be effectively improved by recombination of the internal repeat sequence to form an active circular plasmid [31]. Using PCR and T4 ligase ligation method, the monomeric plasmid could be transformed into multimeric plasmid [32]. However, these methods have obvious disadvantages: (1) Since PCR inevitably introduce unnecessary mutations and it is difficult to perform effective annealing and complementation at both ends of the PCR product, it is not suitable for large-sized plasmid; and (2) Another method based on T4 ligase connection requires a high concentration of monomeric plasmid to ensure the formation of multimeric plasmid. In this study, we exploited the property of PEG4000 to greatly improve the ligation efficiency, and developed a simple and efficient multimer formation method called PAMPL, which not only greatly improved the transformation efficiency of *B. subtilis* SCK6, but also effectively avoided using *Escherichia coli* as an intermediate host.

As a powerful genome editing tool, the CRISPR-Cas9 system has been widely used in many organisms [33]. So far, several CRISPR systems have been developed for genome editing in *B. subtilis* [27, 34–36]. All the plasmid-based CRISPR-Cas system must eliminate previous round of editing plasmids before proceeding to next round of genome editing. However, the traditional plasmid-curing methods include stress-free culture and temperature-sensitive selection, which are time-consuming and labor-intensive. In recent studies, it has been demonstrated that self-targeting gRNA could cure plasmids effectively [14, 37]. To address this problem, we constructed an all-in-one CRISPR-Cas9 system for iterative genome editing based on P_acoR_-gRNA_rep_ curing module in *B. subtilis*. Although the promoter P_acoR_ was regulated by the carbon metabolism activator protein CcpA, the basal leakage activity of the P_acoR_ promoter in CcpA mutant strain was still very low. This indicated that the self-curing module mediated by the P_acoR_ promoter without constraintion of genome context was better than that of the P_spoIVA_ promoter [20]. Therefore, the cell was automatically prepared for the next round of genome editing by using the P_acoR_ promoter to completely inhibit the transcription of gRNA_rep_ in the genome editing and activating P_acoR_ promoter activity during the plasmid curing stage. Based on the excellent characteristics of the system, we anticipate that it can be used to construct *B. subtilis* chassis cells, accelerate the design-build-sequence-learn cycle, and be used for the production of various biomolecules.

## Supplementary Information

**Additional file 1: Table S1.** Primers used in this study. **Fig. S1.** Knockout of *erm* gene using CRISPR-Cas9 gene editing tool. The knockout plasmid pGE-KOerm was transformed into *B. subtilis* SCK6, and the transformants were selected into LB (containing 20 μg/mL tetracycline) liquid medium to induce Cas9 expression. The Cas9 protein cleaves the target site under the guidance of gRNA_erm_, resulting in the DNA double-strand breakage. Homologous recombination templates on bacterial plasmids were used for homologous recombination repair, resulting in a 365 bp base deletion of the *erm* gene. **Fig. S2.** Knockout of *amyE* gene using CRISPR-Cas9 genome editing tool. To further verify the high knockout efficiency of this CRISPR-Cas9 genome editing tool, the gene *amyE* encoding α-amylase was selected. The homologous recombination template on the bacterial plasmid was used for homologous recombination repair, resulting in a 500 bp base deletion of the *amyE* gene. **Fig. S3.** *Spo0A* point mutation and sequencing validation of the point mutation. (a) Introduction of point mutation into *spo0A* gene using CRISPR-Cas9 genome editing tool. Adenine at the position 714 of *spo0A* was converted into thymine, which would result in a stop codon to terminate translation in advance. (b) Sequencing validation of *spo0A* point mutation. The sequencing peak diagram showed that the sequencing result was abnormal, and adenine at the position 714 of *spo0A* was converted into thymine. **Fig. S4.** Insertion of *egfp* gene using CRISPR-Cas9 genome editing tool. The insertion was tested by integration an EGFP cassette into genome.

## Data Availability

All data generated or analyzed during this study are included in this published article and its additional files.

## Abbreviations

LB: Luria–Bertani
ALM: Arabinose-limited medium
TB: Terrific broth
DSM: Difco nutrient broth sporulation medium
Amp: Ampicillin
Erm: Erythromycin
Tet: Tetracycline
PCR: Polymerase chain reaction
*spo0A*: The gene of the central regulatory role in sporulation
*epr*: The gene of minor extracellular protease
*bpr*: The gene of bacillopeptidase F
*amyE*: The gene of α-amylase
SDS-PAGE: Sodium dodecyl sulfate–polyacrylamide gel electrophoresis
PAMPL: PEG4000-assisted monomer plasmid ligation
DFE27: *Douchi* fibrinolytic enzyme DFE27
*erm*: The gene of erythromycin
POE-PCR: Prolonged overlap extension polymerase chain reaction
EGFP: Enhanced green fluorescent protein

## References

[1] Garneau JE, Dupuis ME, Villion M, Romero DA, Barrangou R, Boyaval P, Fremaux C, Horvath P, Magadan AH, Moineau S (2010). The CRISPR/Cas bacterial immune system cleaves bacteriophage and plasmid DNA. Nature.

[2] Rath D, Am Linger L, Rath A, Lundgren M (2015). The CRISPR-Cas immune system: biology, mechanisms and applications. Biochimie.

[3] Deltcheva E, Chylinski K, Sharma CM, Gonzales K, Chao YJ, Pirzada ZA, Eckert MR, Vogel J, Charpentier E (2011). CRISPR RNA maturation by trans-encoded small RNA and host factor RNase III. Nature.

[4] Pyzocha NK, Ran FA, Hsu PD, Zhang F (2014). RNA-guided genome editing of mammalian cells. Methods Mol Bio.

[5] Jiang F, Doudna JA (2017). CRISPR-Cas9 structures and mechanisms. Annu Rev Biophys.

[6] Brouns SJ, Jore MM, Lundgren M, Westra ER, Slijkhuis RJ, Snijders AP, Dickman MJ, Makarova KS, Koonin EV, van der Oost J (2008). Small CRISPR RNAs guide antiviral defense in prokaryotes. Science.

[7] Bowater R, Doherty AJ (2006). Making ends meet: repairing breaks in bacterial DNA by non-homologous end-joining. PLoS Genet.

[8] Cui L, Bikard D (2016). Consequences of Cas9 cleavage in the chromosome of *Escherichia coli*. Nucleic Acids Res.

[9] Zhang K, Su LQ, Duan XD, Liu L, Wu J (2017). High-level extracellular protein production in *Bacillus subtilis* using an optimized dual-promoter expression system. Microb Cell Fact.

[10] Jin P, Kang Z, Yuan PH, Du GC, Chen J (2016). Production of specific-molecular-weight hyaluronan by metabolically engineered *Bacillus subtilis* 168. Metab Eng.

[11] Gu Y, Lv XQ, Liu YF, Li JH, Du GC, Chen J, Rodrigo LA, Liu L (2019). Synthetic redesign of central carbon and redox metabolism for high yield production of N-acetylglucosamine in *Bacillus subtilis*. Metab Eng.

[12] Wu YK, Chen TC, Liu YF, Tian RZ, Lv XQ, Li JH, Du GC, Chen J, Rodrigo LA, Liu L (2020). Design of a programmable biosensor-CRISPRi genetic circuits for dynamic and autonomous dual-control of metabolic flux in *Bacillus subtilis*. Nucleic Acids Res.

[13] Yang HQ, Ma YF, Zhao Y, Shen W, Chen XZ (2020). Systematic engineering of transport and transcription to boost alkaline α-amylase production in *Bacillus subtilis*. Appl Microbiol Biotechnol.

[14] Lim H, Choi SK (2019). Programmed gRNA removal system for CRISPR-Cas9-mediated multi-round genome editing in *Bacillus subtilis*. Front Microbiol..

[15] Wang Y, Liu Y, Liu J, Guo Y, Fan L, Ni X, Zheng X, Wang M, Zheng P, Sun J, Ma Y (2018). MACBETH: multiplex automated *Corynebacterium glutamicum* base editing method. Metab Eng.

[16] Zhang XZ, Zhang YHP (2011). Simple, fast and high-efficiency transformation system for directed evolution of cellulase in *Bacillus subtilis*. Microb Biotechnol.

[17] Wang J, Sui X, Ding Y, Fu Y, Feng X, Liu M, Zhang Y, Xian M, Zhao G (2021). A fast and robust iterative genome-editing method based on a rock-paper-scissors strategy. Nucleic Acids Res.

[18] Wu Y, Liu Y, Lv X, Li J, Du G, Liu L (2020). CAMERS-B: CRISPR/Cpf1 assisted multiple-genes editing and regulation system for *Bacillus subtilis*. Biotechnol Bioeng.

[19] Liu DY, Huang C, Guo JX, Zhang PJ, Chen T, Wang ZW, Zhao XM (2019). Development and characterization of a CRISPR/Cas9n-based multiplex genome editing system for *Bacillus subtilis*. Biotechnol Biofuels.

[20] Silbersack J, Jurgen B, Hecker M, Schneidinger B, Schmuck R, Schweder T (2006). An acetoin-regulated expression system of *Bacillus subtilis*. Appl Microbiol Biotechnol.

[21] Toymentseva AA, Schrecke K, Sharipova MR, Mascher T (2012). The LIKE system, a novel protein expression toolbox for *Bacillus subtilis* based on the liaI promoter. Microb Cell Fact.

[22] Ding T, Huang C, Liang Z, Ma X, Wang N, Huo YX (2020). Reversed paired-gRNA plasmid cloning strategy for efficient genome editing in *Escherichia coli*. Microb Cell Fact.

[23] Fu G, Liu JL, Li JS, Zhu BW, Zhang DW (2018). Systematic screening of optimal signal peptides for secretory production of heterologous proteins in *Bacillus subtilis*. J Agr Food Chem.

[24] You C, Zhang XZ, Zhang YH (2012). Simple cloning via direct transformation of PCR product (DNA Multimer) to *Escherichia coli* and *Bacillus subtilis*. Appl Environ Microbiol.

[25] Cui W, Suo F, Cheng J, Han L, Hao W, Guo J, Zhou Z (2018). Stepwise modifications of genetic parts reinforce the secretory production of nattokinase in *Bacillus subtilis*. Microb Biotechnol.

[26] Westbrook AW, Moo-Young M, Chou CP (2016). Development of a CRISPR-Cas9 tool kit for comprehensive engineering of *Bacillus subtilis*. Appl Environ Microbiol.

[27] Zhang K, Duan X, Wu J (2016). Multigene disruption in undomesticated *Bacillus subtilis* ATCC 6051a using the CRISPR/Cas9 system. Sci Rep..

[28] So Y, Park SY, Park EH, Park SH, Kim EJ, Pan JG, Choi SK (2017). A highly efficient CRISPR-Cas9- mediated large genomic deletion in *Bacillus subtilis*. Front Microbiol.

[29] Michel B, Niaudet B, Ehrlich SD (1982). Intramolecular recombination during plasmid transformation of *Bacillus subtilis* competent cells. EMBO J.

[30] Hong KQ, Liu DY, Chen T, Wang ZW (2018). Recent advances in CRISPR/Cas9 mediated genome editing in *Bacillus subtilis*. World J Microbiol Biotechnol.

[31] Mottes M, Grandi G, Sgaramella V, Canosi U, Morelli G, Trautner TA (1979). Different specific activities of the monomeric and oligomeric forms of plasmid DNA in transformation of *B. subtilis* and *E. coli*. Mol Gen Genet.

[32] Shafikhani S, Siegel RA, Ferrari E, Schellenberger V (1997). Generation of large libraries of random mutants in *Bacillus subtilis* by PCR-based plasmid multimerization. Biotechniques.

[33] Shi S, Qi N, Nielsen J (2021). Microbial production of chemicals driven by CRISPR-Cas systems. Curr Opin Biotechnol.

[34] Altenbuchner J (2016). Editing of the *Bacillus subtilis* genome by the CRISPR-Cas9 system. Appl Environ Microbiol.

[35] So Y, Park SY, Park EH, Park SH, Kim EJ, Pan JG, Choi SK (2017). A highly efficient CRISPR-Cas9-mediated large genomic deletion in *Bacillus subtilis*. Front Microbiol.

[36] Westbrook AW, Moo-Young M, Chou CP (2016). Development of a CRISPR-Cas9 tool kit for comprehensive engineering of *Bacillus subtilis*. Appl Environ Microbiol.

[37] Wu ZW, Wang YJ, Zhang YF, Chen WZ, Wang Y, Ji QJ (2020). Strategies for developing CRISPR-based gene editing methods in bacteria. Small Methods.

