# Supplementary material for: Development and application of a rapid all-in-one plasmid CRISPR-Cas9 system for iterative genome editing in Bacillus subtilis
Source: Microb Cell Fact. 2022 Aug 23;21:173. doi: 10.1186/s12934-022-01896-0 (PMC9400229; doi:10.1186/s12934-022-01896-0)

**Additional file 1**

Development and application of a rapid all-in-one plasmid CRISPR-Cas9 system for iterative genome editing in *Bacillus subtilis*

Yu Zou^1^, Lu Qiu^1^, Aowen Xie ^1^, Wenyuan Han^1^, Shangbo Zhang^1^, Jinshan Li^1, 2^, Shumiao Zhao^1^, Yingjun Li^1^, Yunxiang Liang^1^, Yongmei Hu^1^*

^1^State Key Laboratory of Agricultural Microbiology, College of Life Science and Technology, Huazhong Agricultural University, Wuhan 430070, China

^2^Bioengineering Center, College of Life Science and Technology, Huazhong Agricultural University, Wuhan 430070, China

*Correspondence: plum73@163.com

Tel: +86 27 87281040; Fax: +86 27 87280670

State Key Laboratory of Agricultural Microbiology and College of Life Science and Technology, Huazhong Agricultural University, Wuhan 430070, P.R. China.

**Table S1**. Primers used in this study

| Primers | Sequence（5’-3’） | Description |
| --- | --- | --- |
| P_liaI_-F | CGGGATCCTTTAAAACGCCATGCCTCG | Promoter |
| P_liaI_-R | G**GAATTCATCCTCCTTACGTTT**TCCTTGTCTTCA | P_liaI_ cloning |
| Cas9-F | **AAACGTAAGGAGGATGAATTC**ATGGACAAGAAGTACAGCATCGG | Cas9 gene  Cloning |
| Cas9-R | **CTTTTTTTATAACAGGAATTCT**CAGTCGCCGCCGAGCTG |  |
| mtamp-R | **GCTGCAATGATACCGCGAGA**ACCACGCTCACCGGCTCC | Removing the *Bsa* I restriction |
| mtrepB-F | **ATTTTCTTTTCTCTCCATGG**CCTCACTTTTCCACTTTTTGTCTTG | site |
| mtrepB-R | **CCATGGAGAGAAAAGAAAAT**CGC |  |
| InBsaI-F | TTTCGTTTTATTTGGAAAAAAAGCACCGACTCG | Inserting the *Bsa* I restriction |
| InBsaI-R  Erm-F1  Erm-R1  Erm-F2  Erm-R2 | TGTACAATAGTGATGGAGACCCATATGGGTCTCGTTTTAGAGCTAGAA  CCCAAGCTTtctagggacctctttagctcC  **gatagtgcagtgttgggagt**attccttaccatttaag  **actcccaacactgcactatc**aacacactc  CCCAAGCTTGCTCAGAACCAAATTCCAAGAC | Site  Coustructing the homologous  template |
| amyE-F1 | CCCAAGCTTTGTATTCACTCTGCCAAGTTGT |  |
| amyE-R1 | **AGATATTCGACACCTTGTTCAGTAC**CTAAGTAACGG | Coustructing the homologous |
| amyE-F2 | **GTACTGAACAAGGTGTCGAATATCT**CCCACTATG | template |
| amyE-R2 | CCCAAGCTTCCTGTCAGTTTACCATCGTTC |  |
| spo0A-R1 | CGTTGTTTGGTTAAACTGTC | Coustructing |
| spo0A-F2 | GACAGTTTAACCAAACAACGAGGAAA | the homologous |
| spo0A-R2  P_43_-F  P_43_-R | CCCAAGCTTTAGACGGACTTGCGGTTTTAG  CTGGAATACACAAGGTACCTTATTGAGTGGATGAT  GAAGATCTGTACCGCTATCACTTTATATTTTACA | Template  P_43_ cloning |
| EGFP-F | CGGGATCCTAAGGAGGAGCAATTCATGGTGAGCAAGGGCGAGG | EGFP gene  Cloning |
| EGFP-R  InGFP-R1 | CGGGATCCTTACTTGAGCTCGAGATC  **TCAATAAGGTACCTTGTGTATTCCAG**TCATACAGCC | Coustructing the homologous |
| InGFP-F2 | **CTGGAATACACAAGGTACCTTATTGA**GTGGATGAT | template |

**Table S1**. Primers used in this study (continued)

| Primers | Sequence（5’-3’） | Description |
| --- | --- | --- |
| InGFP-R2 | **GTCATTCAATGGGAATTCCTCG**AGATAAAACGA |  |
| InGFP-F3 | **CGAGGAATTCCCATTGAATGAC**GGGGCAG |  |
| bpr-F1 | CCCAAGCTTGATCAAGGTGAATGGAC |  |
| bpr-R1 | **TGCCTTTTAATGCTCCCAATTC**TTTTCACCTTC |  |
| bpr-F2 | **GAATTGGGAGCATTAAAAGGCA**CGGTCATCAATAAAAC | Coustructing the homologous |
| bpr-R2 | CCCAAGCTTGAGCTTCCGCGTTAAATGG | template |
| epr-F1 | CCCAAGCTTAGTTTTCTCACCATAGGCCC |  |
| epr-R1 | **TCAACGATGTTGGCTCCG**ATAATCCCTGC | Coustructing |
| epr-F2 | **CGGAGCCAACATCGTTGA**TCTTGGTACC | the homologous |
| epr-R2  germ-F  germ-R | CCCAAGCTTTTGTTAAGGCGTTTCTGCAG  AAAACataccgtttacgaaattggaC  tgatGtccaatttcgtaaacggtatG | Template  Targeting  genome |
| gamyE-F | AAAACTGTCTAAGAACCGTTTCAGAC | Targeting |
| gamyE-R | TGATGTCTGAAACGGTTCTTAGACAG | genome |
| gspo0A-F | AAAACCGTTGTTTGGTTATACTGTCC | Targeting |
| gspo0A-R | TGATGGACAGTATAACCAAACAACG | genome |
| gbpr-F | AAAACCTGTGTTTGCAGATGGCTTAC | Targeting |
| gbpr-R | TGATGTAAGCCATCTGCAAACACAGG | genome |
| gepr-F | AAAACCCGGATAATTCACTGGCTTGC | Targeting |
| gepr-R | TGATGCAAGCCAGTGAATTATCCGGG | genome |
| cxerm-F | AACGAATACGGGGGAGATTG | PCR |
| cxerm-R | ACGGAAGTGACGTTTTCCAA | verification |
| cxamyE-F | AAGCGTTCACAGTTTCGG | PCR |
| cxamyE-R | GGATAAAGCACAGCTACAGAC | verification |
| cxspooA-F | AATTGTGAACACACAAGGCT | PCR |
| cxspooA-R | ATCCCGATGTGCTCGTAT | Verification |
| P_glyA_-*Bsa* | CATCGTTTGACTGACTCGAGataaaacgaaaggcc | Synthetic gRNA cassette |
| Ⅰ-*Nde* Ⅰ- *Bsa* | cagtctttcgactgagcctttcgttttatttgGAAA |  |
| Ⅰ-gRNA | AAAAgcaccgactCGGTGCCACTTTTTCAAGTTGA |  |
| scaffold-T1 terminator | TAACGGACTAGCCTTATTTTAacttgctatttctagctctaaaacGAGACCCATATGGGTCTCcatcactattgtacatggtttttaaggccgatgtaaaagattatttttaaaaaatctaaatttttattggtaaaaacgaatattaataacttaaatttaatttaaagttcggaatttatttcactatcttaaaataaaagaaaagactggctatgcagtcctttcattcgcactcataaaccgcccgcactccgccgattagtttcggccgtgtacgcgccaatgtcacatgagctgaaccgaggctgttttgagagacacgcacagggacagccaccggtttcagatgCcaaataaaacgaaaggctcagtcgaaagactgggccttt cgttttat |  |

**Table S1**. Primers used in this study (continued)

| Primers | | Sequence（5’-3’） | | Description |
| --- | --- | --- | --- | --- |
| P_acoR_-gRNA_re_ | CTCGAGTCAGTCAAACGATGCAGAGGaactagga | | Synthetic gRNA cassette | |
| _p_-gRNA | agttaaaaagatttccaaggaaataaatacgtcga | |  |  |
| scaffold | tcattgtcaaaggccgggtgatatccggtcttttttttgcatgctgtaaaacgagacaaatgaatcagtttgagacaaaacgagacacacgtctcaaactgtctccaaagtgaagatgagaagactgattttacgggctcaaaagactggcacacttcttgcatttataatggtgaaccctaaaTAGAAGGAGGCGCACAAAATAATAGAAAATCCATCTTCATGTTTTAGAGCTAGAAATAGCAAGTTAAAATAAGGCTAGTCCGTTATCAACTTGAAAAAGTGGCACCGAGTCGGTGCTTTTTTTGGATCC | |  |  |

Note: The underlined are the restriction site; and the bold are the homologous sequence;

green: promoter; red: gRNA scffold; blue: T1 terminator; orange: spacer


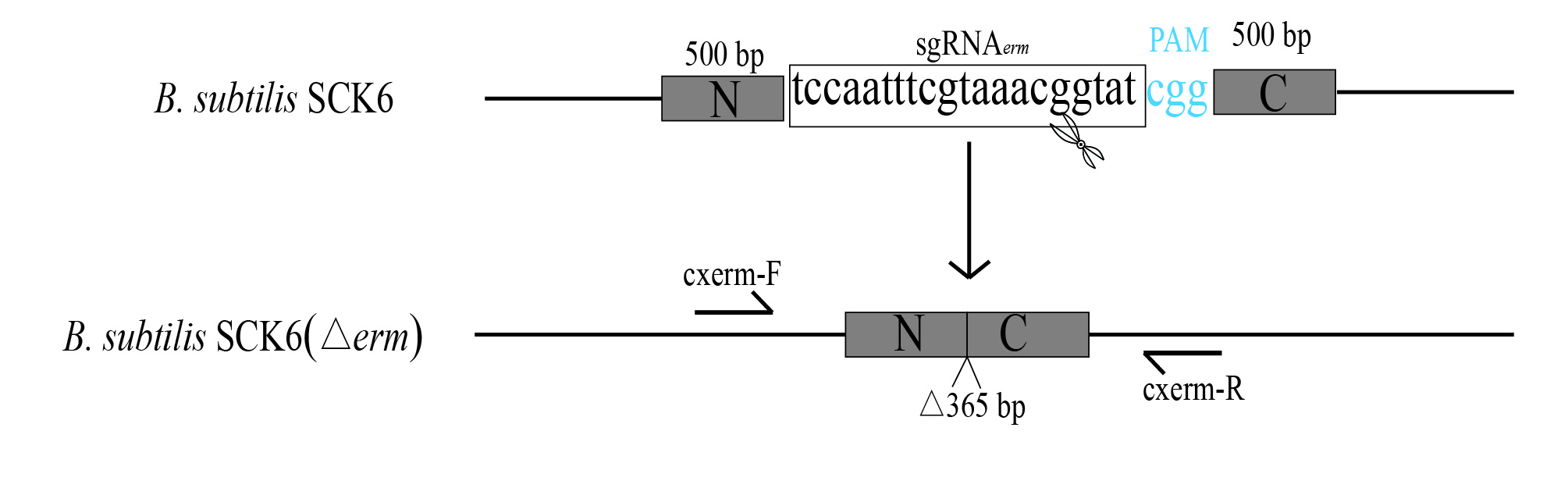


**Fig. S1** Knockout of *erm* gene using CRISPR-Cas9 gene editing tool. The knockout plasmid pGE-KOerm was transformed into *B. subtilis* SCK6, and the transformants were selected into LB (containing 20 μg/mL tetracycline) liquid medium to induce Cas9 expression. The Cas9 protein cleaves the target site under the guidance of gRNA_erm_, resulting in the DNA double-strand breakage. Homologous recombination templates on bacterial plasmids were used for homologous recombination repair, resulting in a 365 bp base deletion of the *erm* gene.


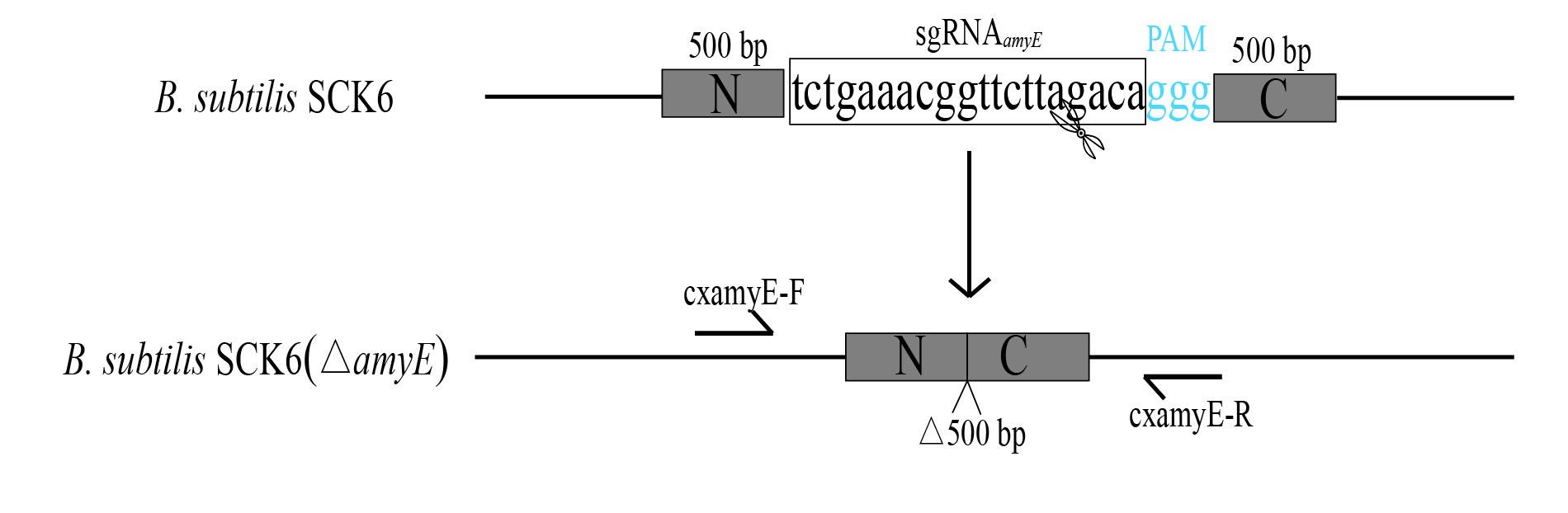


**Fig. S2** Knockout of *amyE* gene using CRISPR-Cas9 gene editing tool. To further verify the high knockout efficiency of this CRISPR-Cas9 genome editing tool, the gene *amyE* encoding α-amylase was selected. The homologous recombination template on the bacterial plasmid was used for homologous recombination repair, resulting in a 500 bp base deletion of the *amyE* gene.


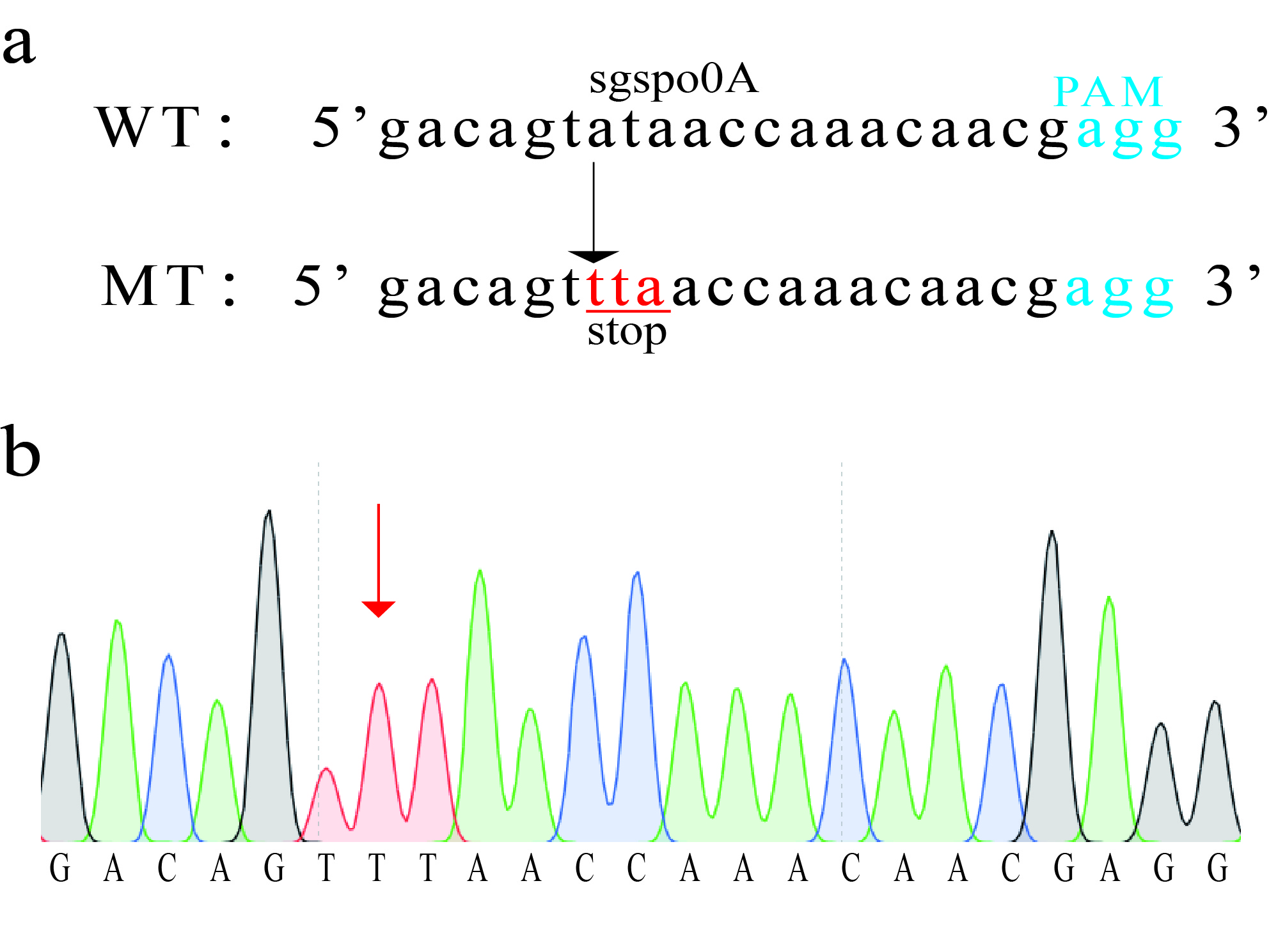


**Fig. S3** *Spo0A* point mutation and sequencing validation of the point mutation. (a) Introduction of point mutation into *spo0A* gene using CRISPR-Cas9 gene editing tool. Adenine at the position 714 of *spo0A* was converted into thymine, which would result in a stop codon to terminate translation in advance. (b) Sequencing validation of *spo0A* point mutation. The sequencing peak diagram showed that the sequencing result was abnormal, and adenine at the position 714 of *spo0A* was converted into thymine.

**Fig. S4** Insertion of *egfp* gene using CRISPR-Cas9 gene editing tool. The insertion was tested by integration an EGFP cassette into genome.


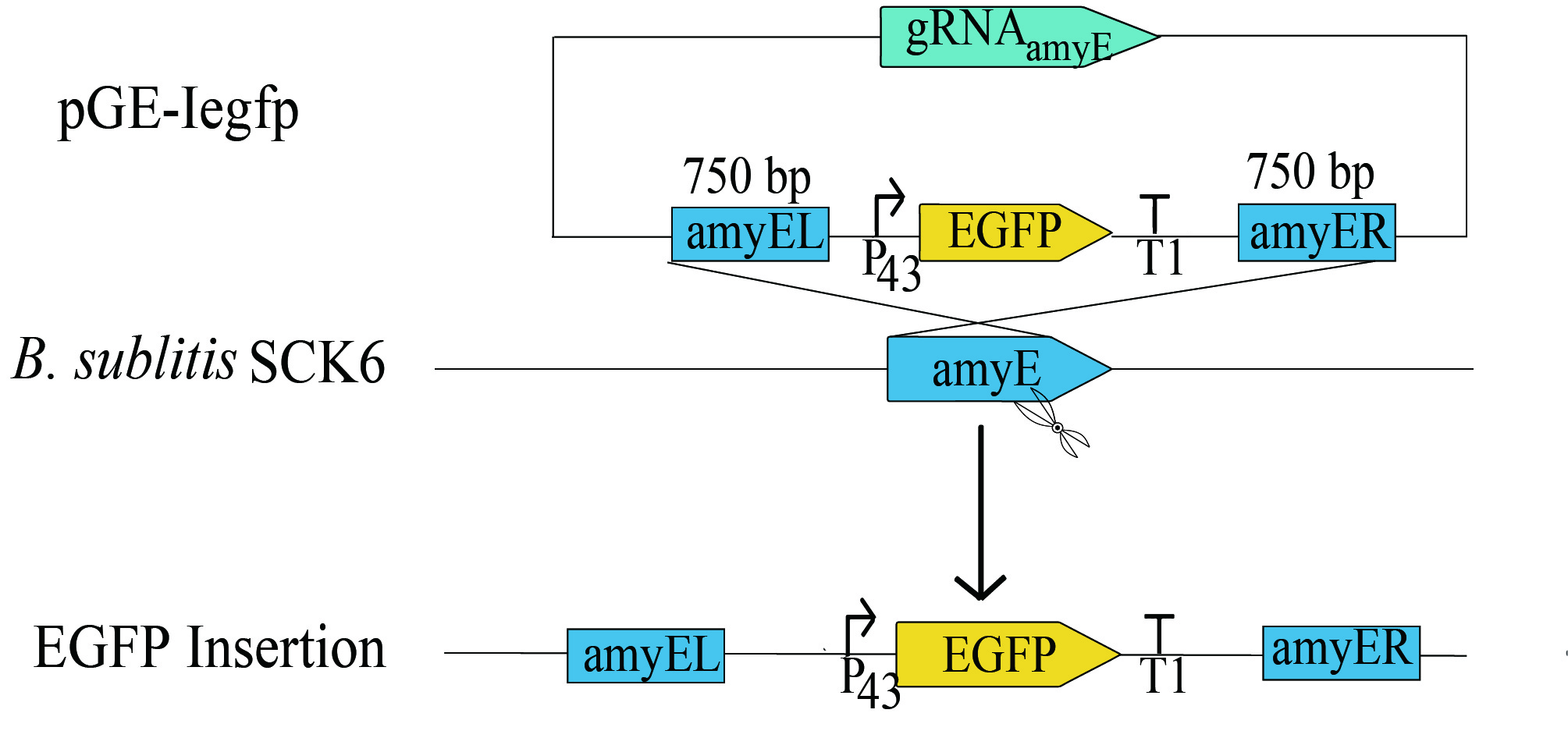

Supplement: Supplementary file 1 — Additional file 1: Table S1. Primers used in this study. Fig. S1. Knockout of erm gene using CRISPR-Cas9 gene editing tool. The knockout plasmid pGE-KOerm was transformed into B. subtilis SCK6, and the transformants were selected into LB (containing 20 μg/mL tetracycline) liquid medium to induce Cas9 expression. The Cas9 protein cleaves the target site under the guidance of gRNAerm, resulting in the DNA double-strand breakage. Homologous recombination templates on bacterial plasmids were used for homologous recombination repair, resulting in a 365 bp base deletion of the erm gene. Fig. S2. Knockout of amyE gene using CRISPR-Cas9 genome editing tool. To further verify the high knockout efficiency of this CRISPR-Cas9 genome editing tool, the gene amyE encoding α-amylase was selected. The homologous recombination template on the bacterial plasmid was used for homologous recombination repair, resulting in a 500 bp base deletion of the amyE gene. Fig. S3. Spo0A point mutation and sequencing validation of the point mutation. (a) Introduction of point mutation into spo0A gene using CRISPR-Cas9 genome editing tool. Adenine at the position 714 of spo0A was converted into thymine, which would result in a stop codon to terminate translation in advance. (b) Sequencing validation of spo0A point mutation. The sequencing peak diagram showed that the sequencing result was abnormal, and adenine at the position 714 of spo0A was converted into thymine. Fig. S4. Insertion of egfp gene using CRISPR-Cas9 genome editing tool. The insertion was tested by integration an EGFP cassette into genome. [file 12934_2022_1896_MOESM1_ESM.docx]
